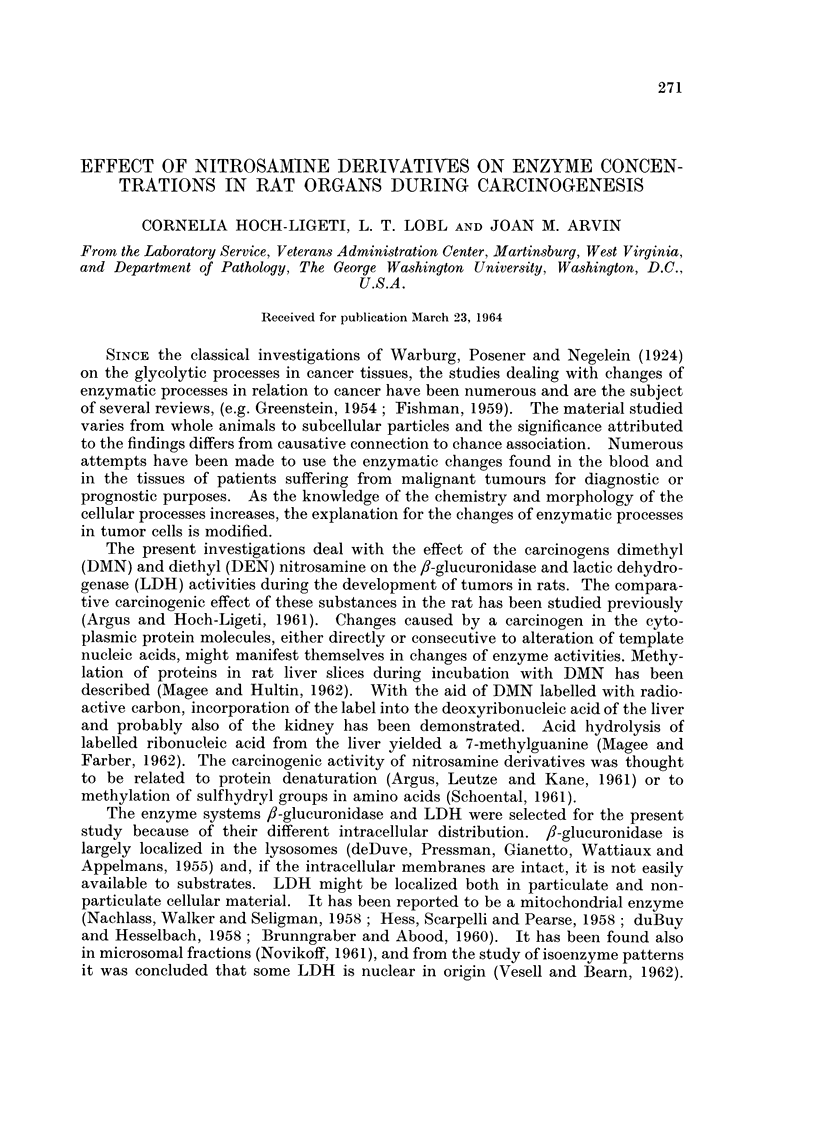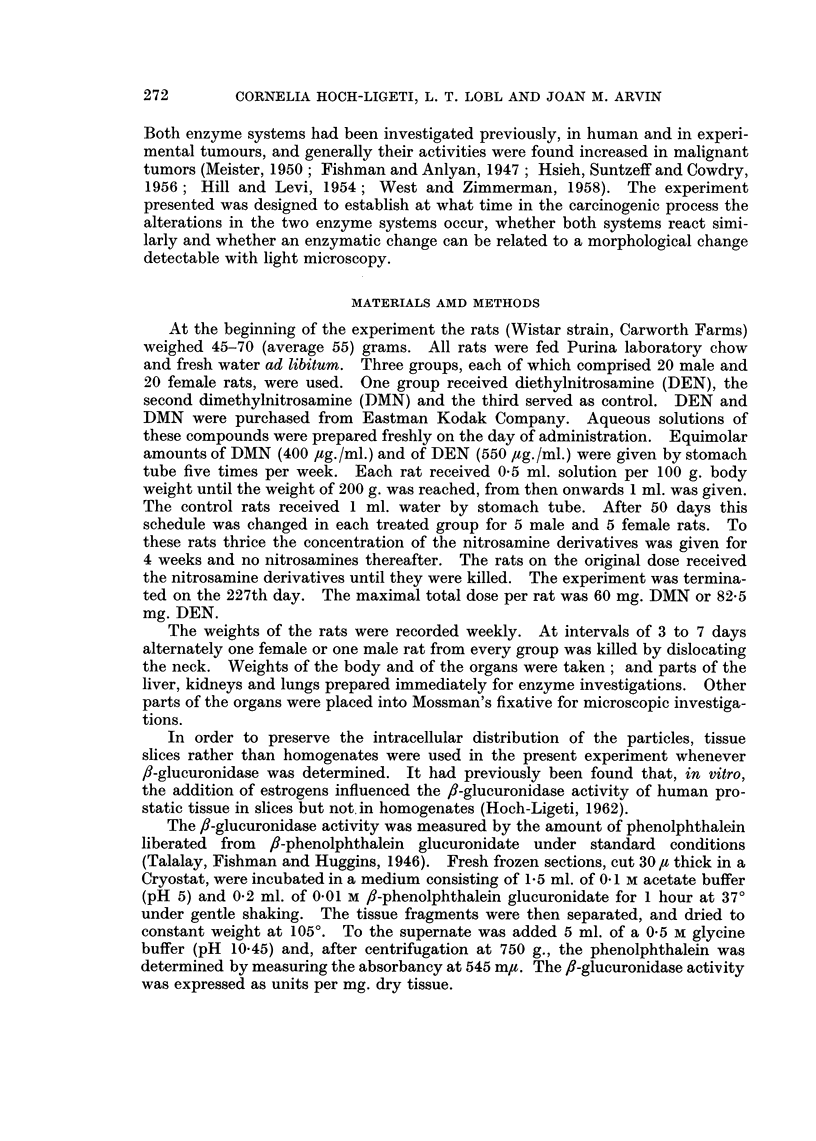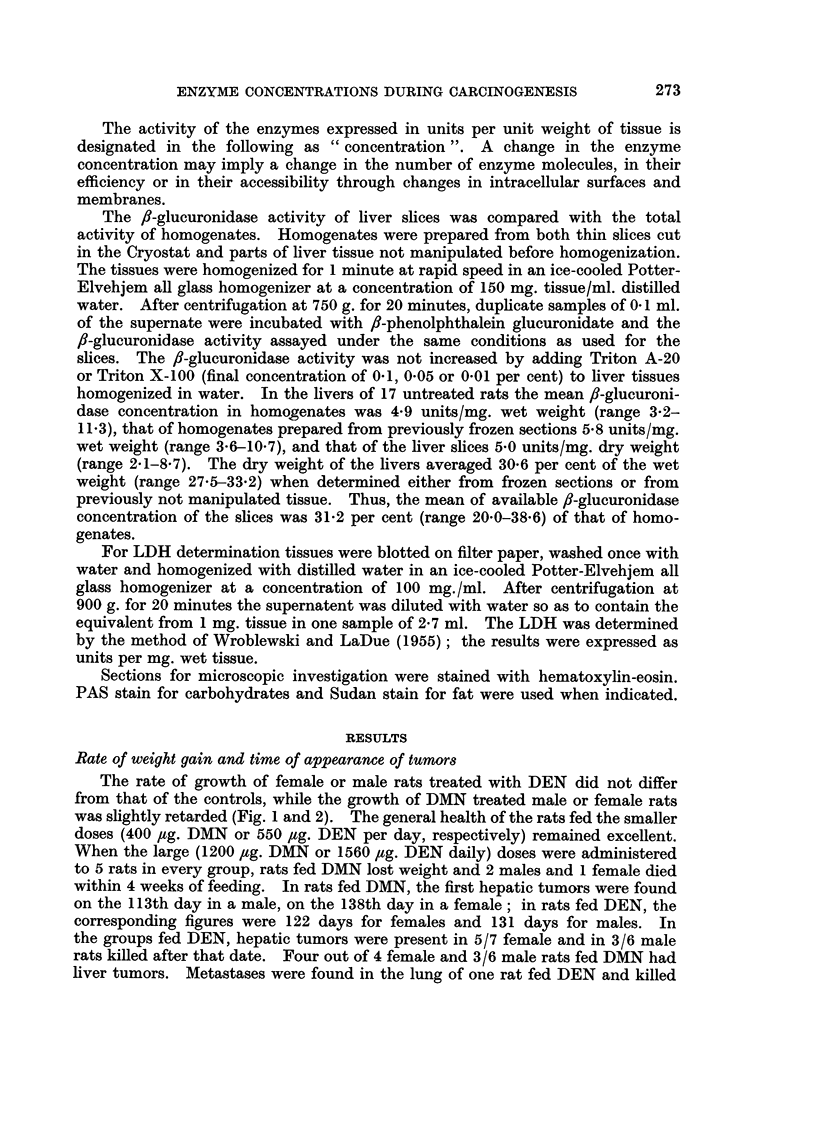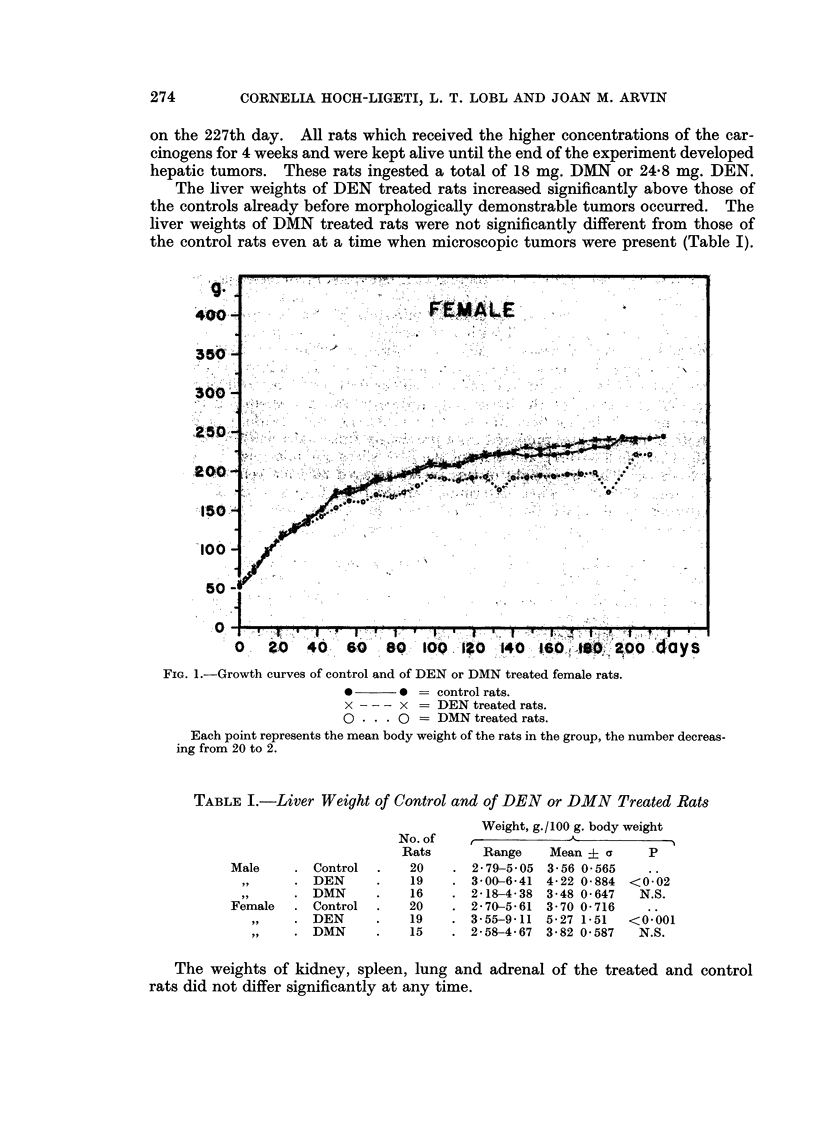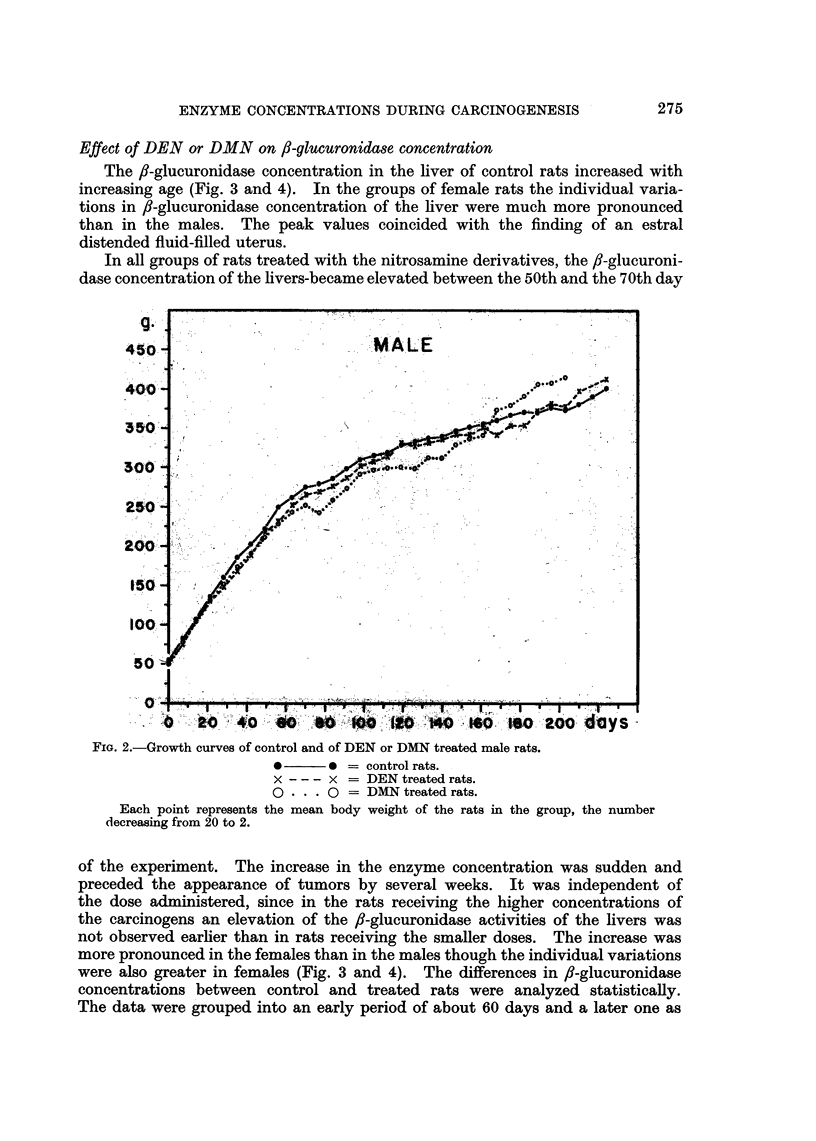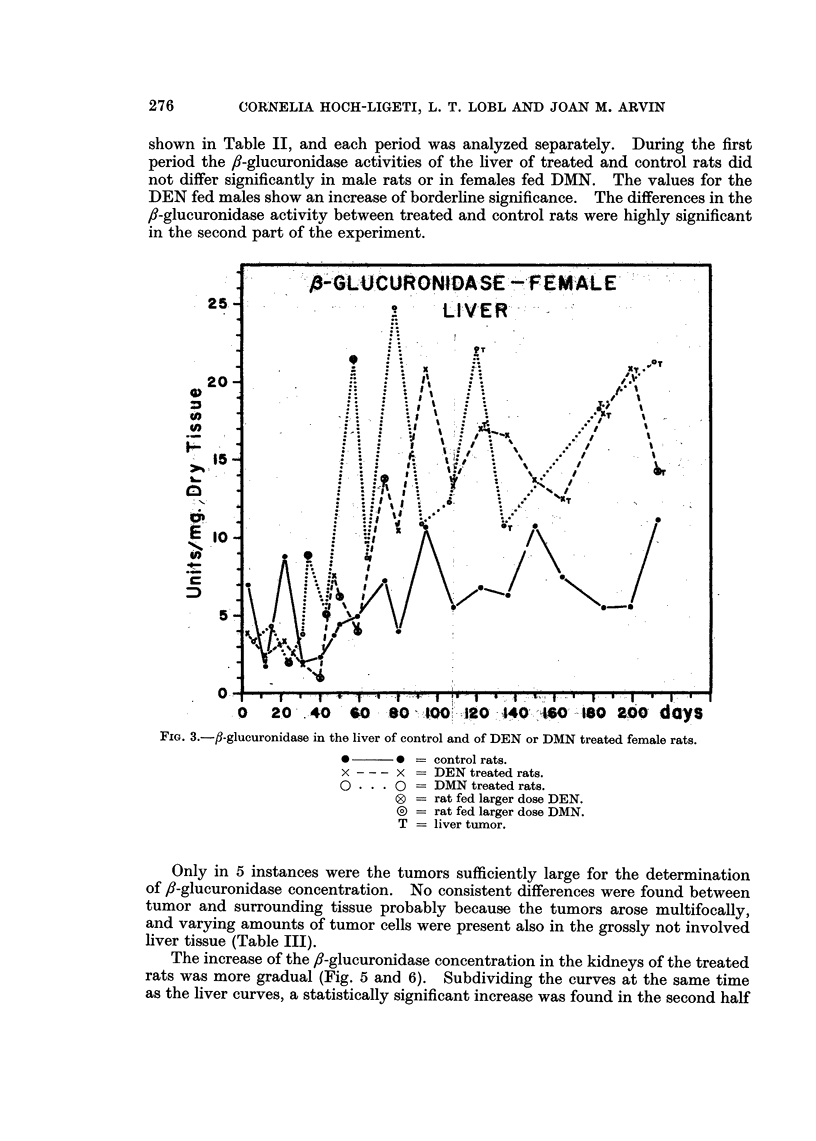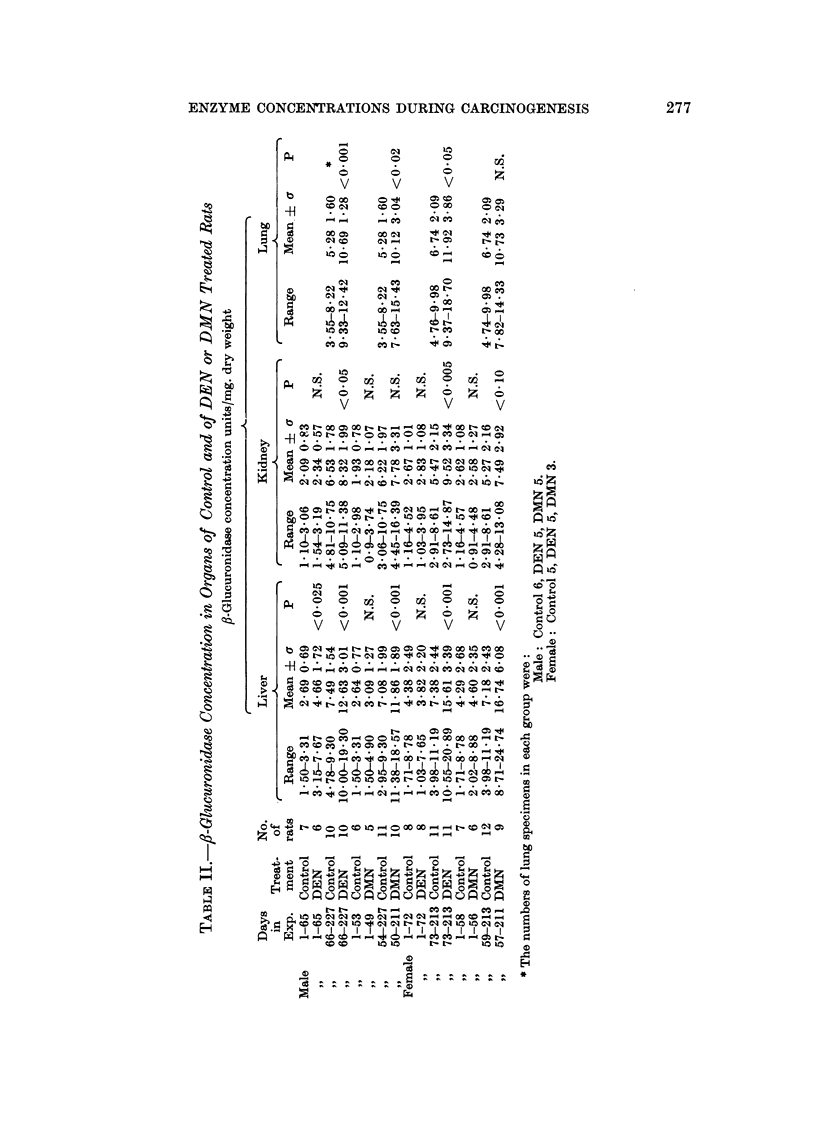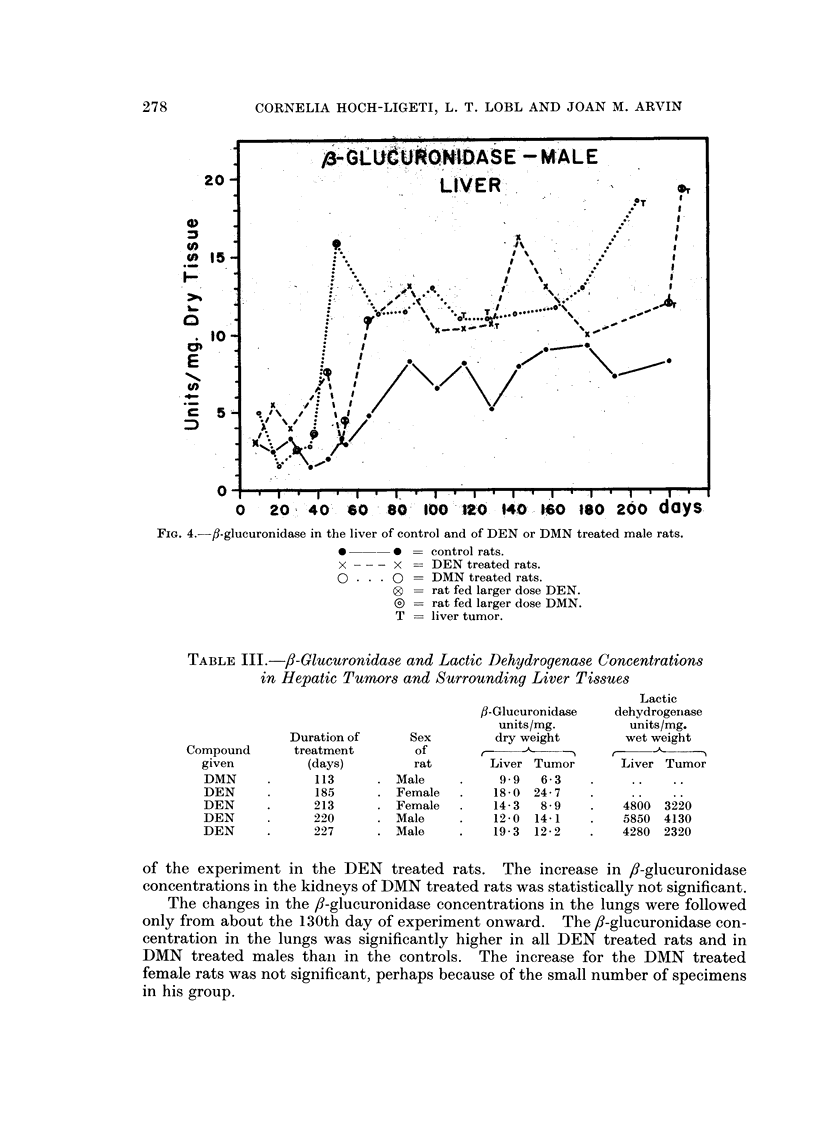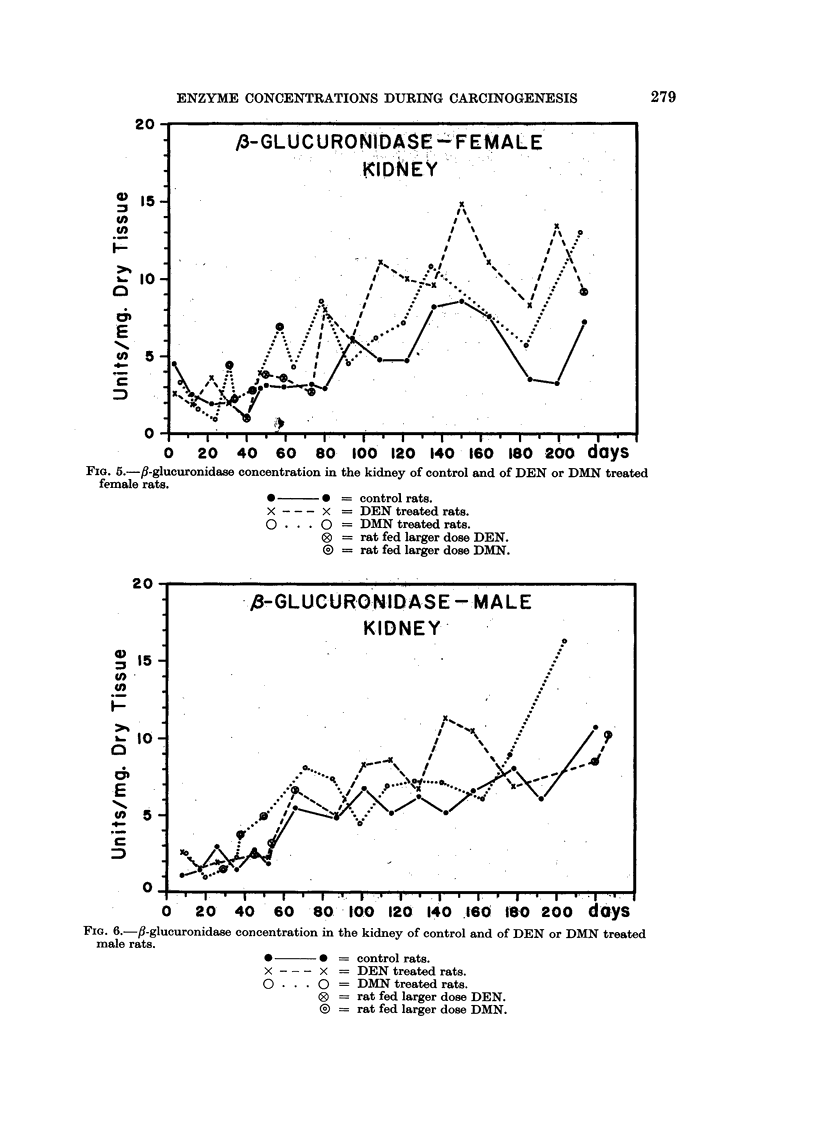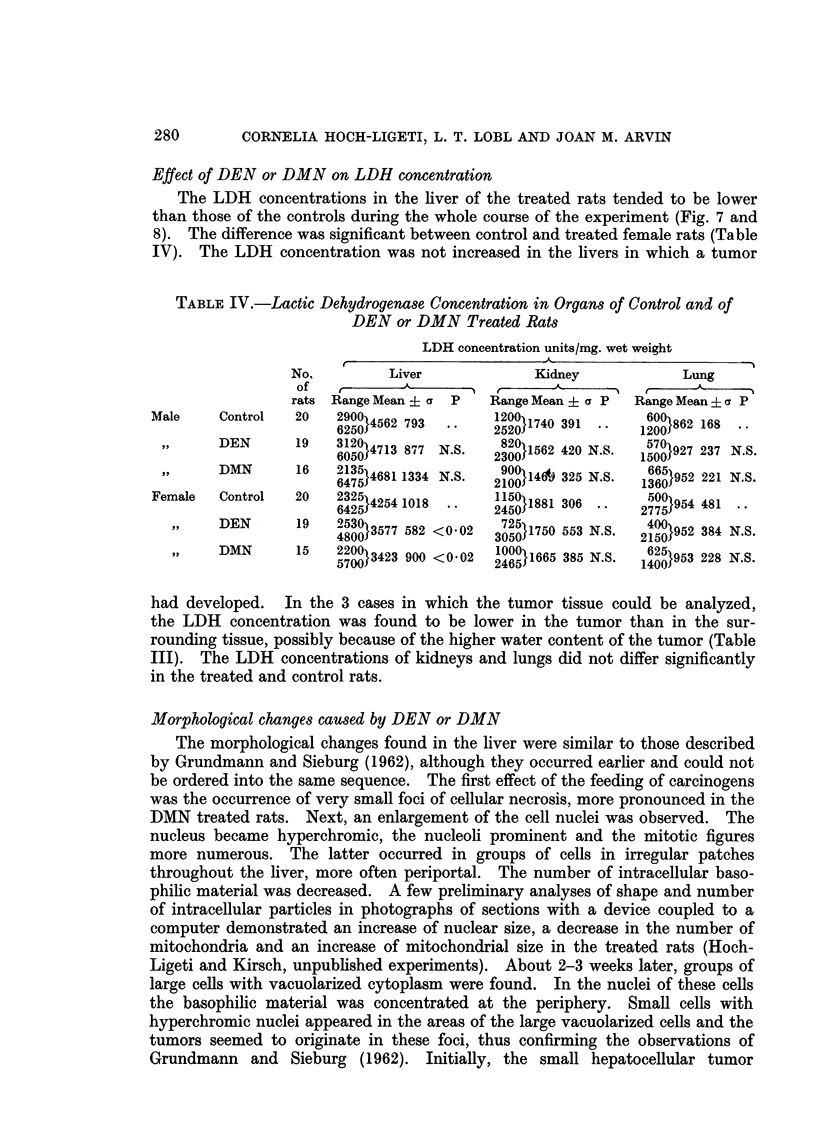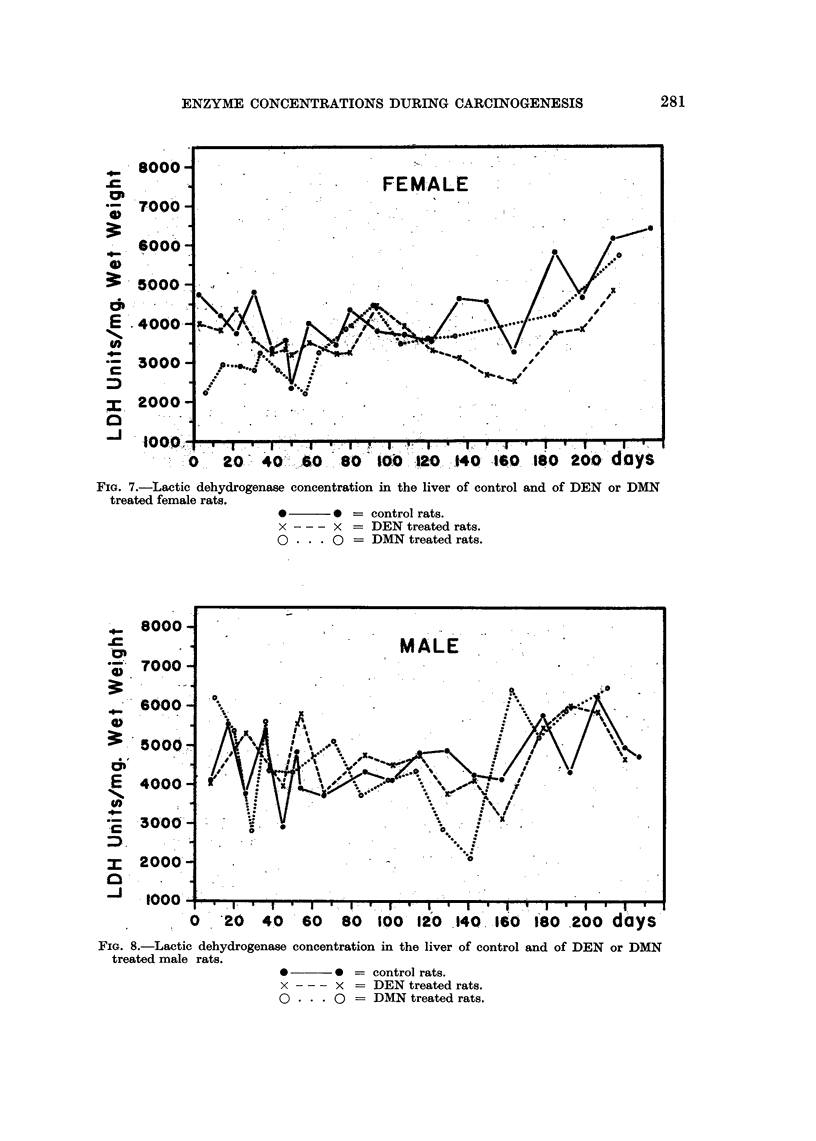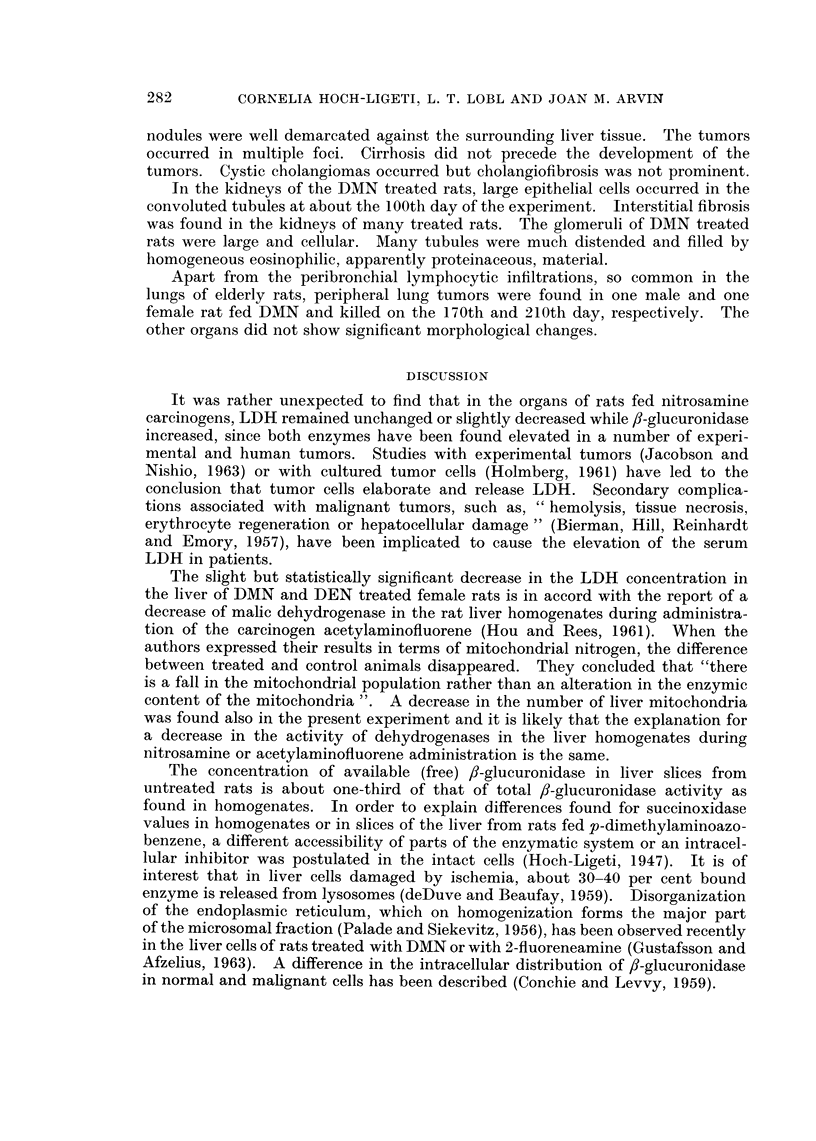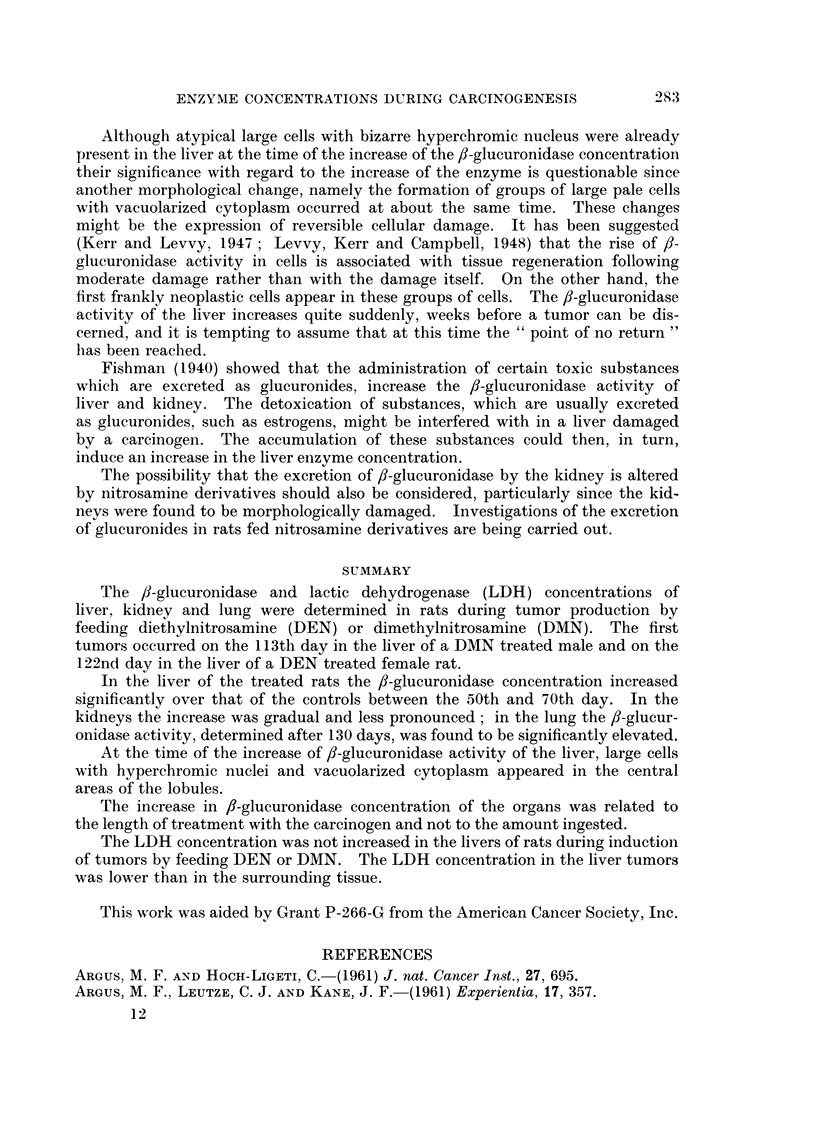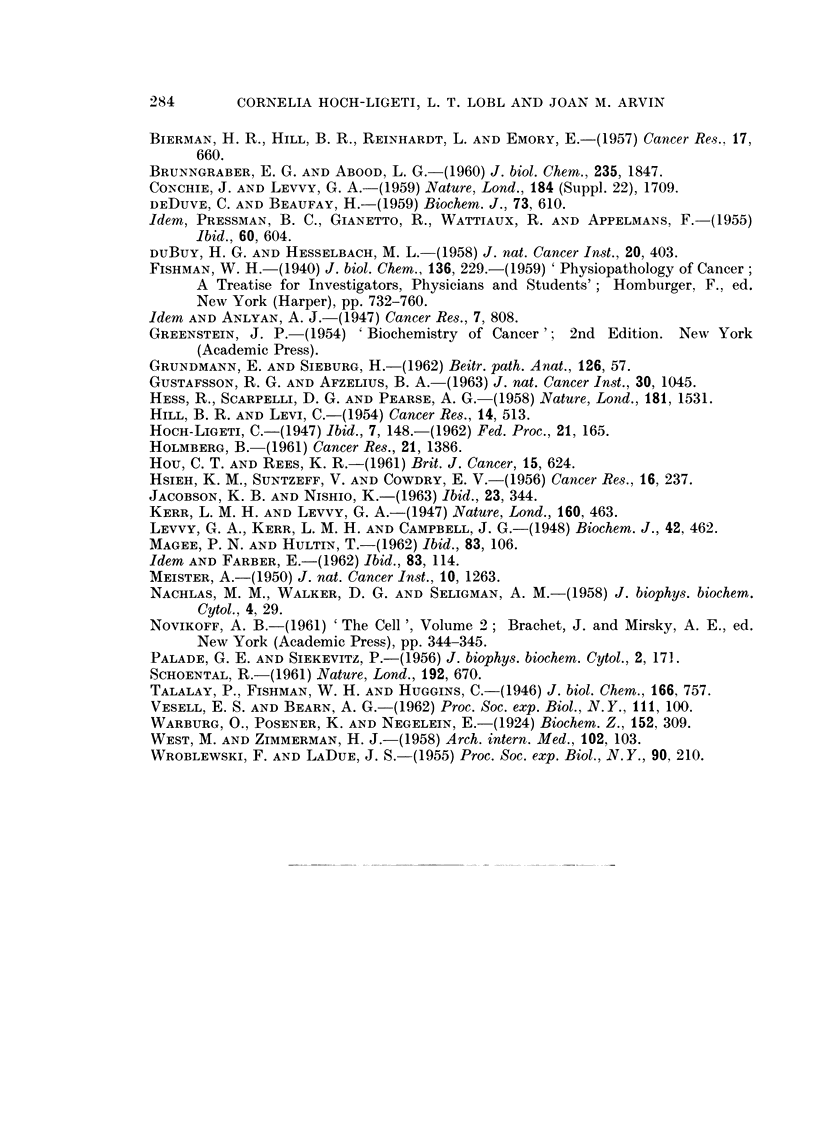# Effect of Nitrosamine Derivatives on Enzyme Concentrations in Rat Organs During Carcinogenesis

**DOI:** 10.1038/bjc.1964.32

**Published:** 1964-06

**Authors:** Cornelia Hoch-Ligeti, L. T. Lobl, Joan M. Arvin


### EFFECT OF NITROSAMINE DERIVATIVES ON ENZYME CONCENTRATIONS IN RAT ORGANS DURING CARCINOGENESIS

CORNELIA HOCH-LIGETI, L. T. LOBL AND JOAN M. ARVIN

*From the Laboratory Service, Veterans Administration Center, Martinsburg, West Virginia, and Department of Pathology, The George Washington University, Washington, D.C., U.S.A.*

Received for publication March 23, 1964

SINCE the classical investigations of Warburg, Posener and Negelein (1924) on the glycolytic processes in cancer tissues, the studies dealing with changes of enzymatic processes in relation to cancer have been numerous and are the subject of several reviews, (e.g. Greenstein, 1954 ; Fishman, 1959). The material studied varies from whole animals to subcellular particles and the significance attributed to the findings differs from causative connection to chance association. Numerous attempts have been made to use the enzymatic changes found in the blood and in the tissues of patients suffering from malignant tumours for diagnostic or prognostic purposes. As the knowledge of the chemistry and morphology of the cellular processes increases, the explanation for the changes of enzymatic processes in tumor cells is modified.

The present investigations deal with the effect of the carcinogens dimethyl (DMN) and diethyl (DEN) nitrosamine on the $\beta$-glucuronidase and lactic dehydrogenase (LDH) activities during the development of tumors in rats. The comparative carcinogenic effect of these substances in the rat has been studied previously (Argus and Hoch-Ligeti, 1961). Changes caused by a carcinogen in the cytoplasmic protein molecules, either directly or consecutive to alteration of template nucleic acids, might manifest themselves in changes of enzyme activities. Methylation of proteins in rat liver slices during incubation with DMN has been described (Magee and Hultin, 1962). With the aid of DMN labelled with radioactive carbon, incorporation of the label into the deoxyribonucleic acid of the liver and probably also of the kidney has been demonstrated. Acid hydrolysis of labelled ribonucleic acid from the liver yielded a 7-methylguanine (Magee and Farber, 1962). The carcinogenic activity of nitrosamine derivatives was thought to be related to protein denaturation (Argus, Leutze and Kane, 1961) or to methylation of sulfhydryl groups in amino acids (Schoental, 1961).

The enzyme systems $\beta$-glucuronidase and LDH were selected for the present study because of their different intracellular distribution. $\beta$-glucuronidase is largely localized in the lysosomes (deDuve, Pressman, Gianetto, Wattiaux and Appelmans, 1955) and, if the intracellular membranes are intact, it is not easily available to substrates. LDH might be localized both in particulate and non-particulate cellular material. It has been reported to be a mitochondrial enzyme (Nachlass, Walker and Seligman, 1958 ; Hess, Scarpelli and Pearse, 1958 ; duBuy and Hesselbach, 1958 ; Brunngraber and Abood, 1960). It has been found also in microsomal fractions (Novikoff, 1961), and from the study of isoenzyme patterns it was concluded that some LDH is nuclear in origin (Vesell and Bearn, 1962).

Both enzyme systems had been investigated previously, in human and in experimental tumours, and generally their activities were found increased in malignant tumors (Meister, 1950 ; Fishman and Anlyan, 1947 ; Hsieh, Suntzeff and Cowdry, 1956 ; Hill and Levi, 1954 ; West and Zimmerman, 1958). The experiment presented was designed to establish at what time in the carcinogenic process the alterations in the two enzyme systems occur, whether both systems react similarly and whether an enzymatic change can be related to a morphological change detectable with light microscopy.

### MATERIALS AMD METHODS

At the beginning of the experiment the rats (Wistar strain, Carworth Farms) weighed 45–70 (average 55) grams. All rats were fed Purina laboratory chow and fresh water *ad libitum*. Three groups, each of which comprised 20 male and 20 female rats, were used. One group received diethylnitrosamine (DEN), the second dimethylnitrosamine (DMN) and the third served as control. DEN and DMN were purchased from Eastman Kodak Company. Aqueous solutions of these compounds were prepared freshly on the day of administration. Equimolar amounts of DMN (400 $\mu$g./ml.) and of DEN (550 $\mu$g./ml.) were given by stomach tube five times per week. Each rat received 0·5 ml. solution per 100 g. body weight until the weight of 200 g. was reached, from then onwards 1 ml. was given. The control rats received 1 ml. water by stomach tube. After 50 days this schedule was changed in each treated group for 5 male and 5 female rats. To these rats thrice the concentration of the nitrosamine derivatives was given for 4 weeks and no nitrosamines thereafter. The rats on the original dose received the nitrosamine derivatives until they were killed. The experiment was terminated on the 227th day. The maximal total dose per rat was 60 mg. DMN or 82·5 mg. DEN.

The weights of the rats were recorded weekly. At intervals of 3 to 7 days alternately one female or one male rat from every group was killed by dislocating the neck. Weights of the body and of the organs were taken ; and parts of the liver, kidneys and lungs prepared immediately for enzyme investigations. Other parts of the organs were placed into Mossman's fixative for microscopic investigations.

In order to preserve the intracellular distribution of the particles, tissue slices rather than homogenates were used in the present experiment whenever $\beta$-glucuronidase was determined. It had previously been found that, *in vitro*, the addition of estrogens influenced the $\beta$-glucuronidase activity of human prostatic tissue in slices but not in homogenates (Hoch-Ligeti, 1962).

The $\beta$-glucuronidase activity was measured by the amount of phenolphthalein liberated from $\beta$-phenolphthalein glucuronidate under standard conditions (Talalay, Fishman and Huggins, 1946). Fresh frozen sections, cut 30 $\mu$ thick in a Cryostat, were incubated in a medium consisting of 1·5 ml. of 0·1 M acetate buffer (pH 5) and 0·2 ml. of 0·01 M $\beta$-phenolphthalein glucuronidate for 1 hour at 37° under gentle shaking. The tissue fragments were then separated, and dried to constant weight at 105°. To the supernate was added 5 ml. of a 0·5 M glycine buffer (pH 10·45) and, after centrifugation at 750 g., the phenolphthalein was determined by measuring the absorbancy at 545 m$\mu$. The $\beta$-glucuronidase activity was expressed as units per mg. dry tissue.

The activity of the enzymes expressed in units per unit weight of tissue is designated in the following as " concentration ". A change in the enzyme concentration may imply a change in the number of enzyme molecules, in their efficiency or in their accessibility through changes in intracellular surfaces and membranes.

The $\beta$-glucuronidase activity of liver slices was compared with the total activity of homogenates. Homogenates were prepared from both thin slices cut in the Cryostat and parts of liver tissue not manipulated before homogenization. The tissues were homogenized for 1 minute at rapid speed in an ice-cooled Potter-Elvehjem all glass homogenizer at a concentration of 150 mg. tissue/ml. distilled water. After centrifugation at 750 g. for 20 minutes, duplicate samples of 0·1 ml. of the supernate were incubated with $\beta$-phenolphthalein glucuronidate and the $\beta$-glucuronidase activity assayed under the same conditions as used for the slices. The $\beta$-glucuronidase activity was not increased by adding Triton A-20 or Triton X-100 (final concentration of 0·1, 0·05 or 0·01 per cent) to liver tissues homogenized in water. In the livers of 17 untreated rats the mean $\beta$-glucuronidase concentration in homogenates was 4·9 units/mg. wet weight (range 3·2–11·3), that of homogenates prepared from previously frozen sections 5·8 units/mg. wet weight (range 3·6–10·7), and that of the liver slices 5·0 units/mg. dry weight (range 2·1–8·7). The dry weight of the livers averaged 30·6 per cent of the wet weight (range 27·5–33·2) when determined either from frozen sections or from previously not manipulated tissue. Thus, the mean of available $\beta$-glucuronidase concentration of the slices was 31·2 per cent (range 20·0–38·6) of that of homogenates.

For LDH determination tissues were blotted on filter paper, washed once with water and homogenized with distilled water in an ice-cooled Potter-Elvehjem all glass homogenizer at a concentration of 100 mg./ml. After centrifugation at 900 g. for 20 minutes the supernatent was diluted with water so as to contain the equivalent from 1 mg. tissue in one sample of 2·7 ml. The LDH was determined by the method of Wroblewski and LaDue (1955) ; the results were expressed as units per mg. wet tissue.

Sections for microscopic investigation were stained with hematoxylin-eosin. PAS stain for carbohydrates and Sudan stain for fat were used when indicated.

### RESULTS

### *Rate of weight gain and time of appearance of tumors*

The rate of growth of female or male rats treated with DEN did not differ from that of the controls, while the growth of DMN treated male or female rats was slightly retarded (Fig. 1 and 2). The general health of the rats fed the smaller doses (400 $\mu$g. DMN or 550 $\mu$g. DEN per day, respectively) remained excellent. When the large (1200 $\mu$g. DMN or 1560 $\mu$g. DEN daily) doses were administered to 5 rats in every group, rats fed DMN lost weight and 2 males and 1 female died within 4 weeks of feeding. In rats fed DMN, the first hepatic tumors were found on the 113th day in a male, on the 138th day in a female ; in rats fed DEN, the corresponding figures were 122 days for females and 131 days for males. In the groups fed DEN, hepatic tumors were present in 5/7 female and in 3/6 male rats killed after that date. Four out of 4 female and 3/6 male rats fed DMN had liver tumors. Metastases were found in the lung of one rat fed DEN and killed on the 227th day. All rats which received the higher concentrations of the carcinogens for 4 weeks and were kept alive until the end of the experiment developed hepatic tumors. These rats ingested a total of 18 mg. DMN or 24·8 mg. DEN.

The liver weights of DEN treated rats increased significantly above those of the controls already before morphologically demonstrable tumors occurred. The liver weights of DMN treated rats were not significantly different from those of the control rats even at a time when microscopic tumors were present (Table I).

FIG. 1.—Growth curves of control and of DEN or DMN treated female rats.

● ——— ● = control rats.
× – – – × = DEN treated rats.
○ . . . ○ = DMN treated rats.

Each point represents the mean body weight of the rats in the group, the number decreasing from 20 to 2.

TABLE I.—*Liver Weight of Control and of DEN or DMN Treated Rats*

| | | No. of Rats | Weight, g./100 g. body weight: Range | Mean ± σ | | P |
|---|---|---|---|---|---|---|
| Male | Control | 20 | 2·79–5·05 | 3·56 | 0·565 | . . |
| ,, | DEN | 19 | 3·00–6·41 | 4·22 | 0·884 | <0·02 |
| ,, | DMN | 16 | 2·18–4·38 | 3·48 | 0·647 | N.S. |
| Female | Control | 20 | 2·70–5·61 | 3·70 | 0·716 | . . |
| ,, | DEN | 19 | 3·55–9·11 | 5·27 | 1·51 | <0·001 |
| ,, | DMN | 15 | 2·58–4·67 | 3·82 | 0·587 | N.S. |

The weights of kidney, spleen, lung and adrenal of the treated and control rats did not differ significantly at any time.

*Effect of DEN or DMN on β-glucuronidase concentration*

The β-glucuronidase concentration in the liver of control rats increased with increasing age (Fig. 3 and 4). In the groups of female rats the individual variations in β-glucuronidase concentration of the liver were much more pronounced than in the males. The peak values coincided with the finding of an estral distended fluid-filled uterus.

In all groups of rats treated with the nitrosamine derivatives, the β-glucuronidase concentration of the livers-became elevated between the 50th and the 70th day

FIG. 2.—Growth curves of control and of DEN or DMN treated male rats.

● ——— ● = control rats.
× - - - × = DEN treated rats.
○ . . . ○ = DMN treated rats.

Each point represents the mean body weight of the rats in the group, the number decreasing from 20 to 2.

of the experiment. The increase in the enzyme concentration was sudden and preceded the appearance of tumors by several weeks. It was independent of the dose administered, since in the rats receiving the higher concentrations of the carcinogens an elevation of the β-glucuronidase activities of the livers was not observed earlier than in rats receiving the smaller doses. The increase was more pronounced in the females than in the males though the individual variations were also greater in females (Fig. 3 and 4). The differences in β-glucuronidase concentrations between control and treated rats were analyzed statistically. The data were grouped into an early period of about 60 days and a later one as shown in Table II, and each period was analyzed separately. During the first period the β-glucuronidase activities of the liver of treated and control rats did not differ significantly in male rats or in females fed DMN. The values for the DEN fed males show an increase of borderline significance. The differences in the β-glucuronidase activity between treated and control rats were highly significant in the second part of the experiment.

FIG. 3.—β-glucuronidase in the liver of control and of DEN or DMN treated female rats.

●——● = control rats.
× – – – × = DEN treated rats.
○ . . . ○ = DMN treated rats.
⊗ = rat fed larger dose DEN.
◎ = rat fed larger dose DMN.
T = liver tumor.

Only in 5 instances were the tumors sufficiently large for the determination of β-glucuronidase concentration. No consistent differences were found between tumor and surrounding tissue probably because the tumors arose multifocally, and varying amounts of tumor cells were present also in the grossly not involved liver tissue (Table III).

The increase of the β-glucuronidase concentration in the kidneys of the treated rats was more gradual (Fig. 5 and 6). Subdividing the curves at the same time as the liver curves, a statistically significant increase was found in the second half

TABLE II.—*β-Glucuronidase Concentration in Organs of Control and of DEN or DMN Treated Rats*

| | Days in Exp. | Treatment | No. of rats | Liver Range | Liver Mean ± σ | Liver P | Kidney Range | Kidney Mean ± σ | Kidney P | Lung Range | Lung Mean ± σ | Lung P |
|---|---|---|---|---|---|---|---|---|---|---|---|---|
| | | | | β-Glucuronidase concentration units/mg. dry weight | | | | | | | | |
| Male | 1–65 | Control | 7 | 1·50–3·31 | 2·69 0·69 | | 1·10–3·06 | 2·09 0·83 | | | | |
| ,, | 1–65 | DEN | 6 | 3·15–7·67 | 4·66 1·72 | <0·025 | 1·54–3·19 | 2·34 0·57 | N.S. | | | |
| ,, | 66–227 | Control | 10 | 4·78–9·30 | 7·49 1·54 | | 4·81–10·75 | 6·53 1·78 | | 3·55–8·22 | 5·28 1·60 | * |
| ,, | 66–227 | DEN | 10 | 10·00–19·30 | 12·63 3·01 | <0·001 | 5·09–11·38 | 8·32 1·99 | <0·05 | 9·33–12·42 | 10·69 1·28 | <0·001 |
| ,, | 1–53 | Control | 6 | 1·50–3·31 | 2·64 0·77 | | 1·10–2·98 | 1·93 0·78 | | | | |
| ,, | 1–49 | DMN | 5 | 1·50–4·90 | 3·09 1·27 | N.S. | 0·9–3·74 | 2·18 1·07 | N.S. | | | |
| ,, | 54–227 | Control | 11 | 2·95–9·30 | 7·08 1·99 | | 3·06–10·75 | 6·22 1·97 | | 3·55–8·22 | 5·28 1·60 | |
| ,, | 50–211 | DMN | 10 | 11·38–18·57 | 11·86 1·89 | <0·001 | 4·45–16·39 | 7·78 3·31 | N.S. | 7·63–15·43 | 10·12 3·04 | <0·02 |
| Female | 1–72 | Control | 8 | 1·71–8·78 | 4·38 2·49 | | 1·16–4·52 | 2·67 1·01 | | | | |
| ,, | 1–72 | DEN | 8 | 1·03–7·65 | 3·82 2·20 | N.S. | 1·03–3·95 | 2·83 1·08 | N.S. | | | |
| ,, | 73–213 | Control | 11 | 3·98–11·19 | 7·38 2·44 | | 2·91–8·61 | 5·47 2·15 | | 4·76–9·98 | 6·74 2·09 | |
| ,, | 73–213 | DEN | 11 | 10·55–20·89 | 15·61 3·39 | <0·001 | 2·73–14·87 | 9·52 3·34 | <0·005 | 9·37–18·70 | 11·92 3·86 | <0·05 |
| ,, | 1–58 | Control | 7 | 1·71–8·78 | 4·29 2·68 | | 1·16–4·57 | 2·62 1·08 | | | | |
| ,, | 1–56 | DMN | 6 | 2·02–8·88 | 4·60 2·35 | N.S. | 0·91–4·48 | 2·58 1·27 | N.S. | | | |
| ,, | 59–213 | Control | 12 | 3·98–11·19 | 7·18 2·43 | | 2·91–8·61 | 5·27 2·16 | | 4·74–9·98 | 6·74 2·09 | |
| ,, | 57–211 | DMN | 9 | 8·71–24·74 | 16·74 6·08 | <0·001 | 4·28–13·08 | 7·49 2·92 | <0·10 | 7·82–14·33 | 10·73 3·29 | N.S. |

* The numbers of lung specimens in each group were :

Male : Control 6, DEN 5, DMN 5.

Female : Control 5, DEN 5, DMN 3.

FIG. 4.—β-glucuronidase in the liver of control and of DEN or DMN treated male rats.

● ——— ● = control rats.
× - - - × = DEN treated rats.
○ . . . ○ = DMN treated rats.
⊗ = rat fed larger dose DEN.
◎ = rat fed larger dose DMN.
T = liver tumor.

TABLE III.—*β-Glucuronidase and Lactic Dehydrogenase Concentrations in Hepatic Tumors and Surrounding Liver Tissues*

| Compound given | Duration of treatment (days) | Sex of rat | β-Glucuronidase units/mg. dry weight | | Lactic dehydrogenase units/mg. wet weight | |
|---|---|---|---|---|---|---|
| | | | Liver | Tumor | Liver | Tumor |
| DMN | 113 | Male | 9·9 | 6·3 | . . | . . |
| DEN | 185 | Female | 18·0 | 24·7 | . . | . . |
| DEN | 213 | Female | 14·3 | 8·9 | 4800 | 3220 |
| DEN | 220 | Male | 12·0 | 14·1 | 5850 | 4130 |
| DEN | 227 | Male | 19·3 | 12·2 | 4280 | 2320 |

of the experiment in the DEN treated rats. The increase in β-glucuronidase concentrations in the kidneys of DMN treated rats was statistically not significant.

The changes in the β-glucuronidase concentrations in the lungs were followed only from about the 130th day of experiment onward. The β-glucuronidase concentration in the lungs was significantly higher in all DEN treated rats and in DMN treated males than in the controls. The increase for the DMN treated female rats was not significant, perhaps because of the small number of specimens in his group.

FIG. 5.—β-glucuronidase concentration in the kidney of control and of DEN or DMN treated female rats.

●——● = control rats.
× - - - × = DEN treated rats.
○ . . . ○ = DMN treated rats.
⊗ = rat fed larger dose DEN.
◎ = rat fed larger dose DMN.

FIG. 6.—β-glucuronidase concentration in the kidney of control and of DEN or DMN treated male rats.

●——● = control rats.
× - - - × = DEN treated rats.
○ . . . ○ = DMN treated rats.
⊗ = rat fed larger dose DEN.
◎ = rat fed larger dose DMN.

*Effect of DEN or DMN on LDH concentration*

The LDH concentrations in the liver of the treated rats tended to be lower than those of the controls during the whole course of the experiment (Fig. 7 and 8). The difference was significant between control and treated female rats (Table IV). The LDH concentration was not increased in the livers in which a tumor had developed. In the 3 cases in which the tumor tissue could be analyzed, the LDH concentration was found to be lower in the tumor than in the surrounding tissue, possibly because of the higher water content of the tumor (Table III). The LDH concentrations of kidneys and lungs did not differ significantly in the treated and control rats.

TABLE IV.—*Lactic Dehydrogenase Concentration in Organs of Control and of DEN or DMN Treated Rats*

| | | | LDH concentration units/mg. wet weight | | | | | | | | | | | |
|---|---|---|---|---|---|---|---|---|---|---|---|---|---|---|
| | | No. of rats | Liver | | | | Kidney | | | | Lung | | | |
| | | | Range | Mean | ± σ | P | Range | Mean | ± σ | P | Range | Mean | ± σ | P |
| Male | Control | 20 | 2900–6250 | 4562 | 793 | .. | 1200–2520 | 1740 | 391 | .. | 600–1200 | 862 | 168 | .. |
| ” | DEN | 19 | 3120–6050 | 4713 | 877 | N.S. | 820–2300 | 1562 | 420 | N.S. | 570–1500 | 927 | 237 | N.S. |
| ” | DMN | 16 | 2135–6475 | 4681 | 1334 | N.S. | 900–2100 | 1469 | 325 | N.S. | 665–1360 | 952 | 221 | N.S. |
| Female | Control | 20 | 2325–6425 | 4254 | 1018 | .. | 1150–2450 | 1881 | 306 | .. | 500–2775 | 954 | 481 | .. |
| ” | DEN | 19 | 2530–4800 | 3577 | 582 | $<0\cdot02$ | 725–3050 | 1750 | 553 | N.S. | 400–2150 | 952 | 384 | N.S. |
| ” | DMN | 15 | 2200–5700 | 3423 | 900 | $<0\cdot02$ | 1000–2465 | 1665 | 385 | N.S. | 625–1400 | 953 | 228 | N.S. |

*Morphological changes caused by DEN or DMN*

The morphological changes found in the liver were similar to those described by Grundmann and Sieburg (1962), although they occurred earlier and could not be ordered into the same sequence. The first effect of the feeding of carcinogens was the occurrence of very small foci of cellular necrosis, more pronounced in the DMN treated rats. Next, an enlargement of the cell nuclei was observed. The nucleus became hyperchromic, the nucleoli prominent and the mitotic figures more numerous. The latter occurred in groups of cells in irregular patches throughout the liver, more often periportal. The number of intracellular basophilic material was decreased. A few preliminary analyses of shape and number of intracellular particles in photographs of sections with a device coupled to a computer demonstrated an increase of nuclear size, a decrease in the number of mitochondria and an increase of mitochondrial size in the treated rats (Hoch-Ligeti and Kirsch, unpublished experiments). About 2–3 weeks later, groups of large cells with vacuolarized cytoplasm were found. In the nuclei of these cells the basophilic material was concentrated at the periphery. Small cells with hyperchromic nuclei appeared in the areas of the large vacuolarized cells and the tumors seemed to originate in these foci, thus confirming the observations of Grundmann and Sieburg (1962). Initially, the small hepatocellular tumor

FIG. 7.—Lactic dehydrogenase concentration in the liver of control and of DEN or DMN treated female rats.

●——● = control rats.
× - - - × = DEN treated rats.
○ . . . ○ = DMN treated rats.

FIG. 8.—Lactic dehydrogenase concentration in the liver of control and of DEN or DMN treated male rats.

●——● = control rats.
× - - - × = DEN treated rats.
○ . . . ○ = DMN treated rats.

nodules were well demarcated against the surrounding liver tissue. The tumors occurred in multiple foci. Cirrhosis did not precede the development of the tumors. Cystic cholangiomas occurred but cholangiofibrosis was not prominent.

In the kidneys of the DMN treated rats, large epithelial cells occurred in the convoluted tubules at about the 100th day of the experiment. Interstitial fibrosis was found in the kidneys of many treated rats. The glomeruli of DMN treated rats were large and cellular. Many tubules were much distended and filled by homogeneous eosinophilic, apparently proteinaceous, material.

Apart from the peribronchial lymphocytic infiltrations, so common in the lungs of elderly rats, peripheral lung tumors were found in one male and one female rat fed DMN and killed on the 170th and 210th day, respectively. The other organs did not show significant morphological changes.

### DISCUSSION

It was rather unexpected to find that in the organs of rats fed nitrosamine carcinogens, LDH remained unchanged or slightly decreased while $\beta$-glucuronidase increased, since both enzymes have been found elevated in a number of experimental and human tumors. Studies with experimental tumors (Jacobson and Nishio, 1963) or with cultured tumor cells (Holmberg, 1961) have led to the conclusion that tumor cells elaborate and release LDH. Secondary complications associated with malignant tumors, such as, " hemolysis, tissue necrosis, erythrocyte regeneration or hepatocellular damage " (Bierman, Hill, Reinhardt and Emory, 1957), have been implicated to cause the elevation of the serum LDH in patients.

The slight but statistically significant decrease in the LDH concentration in the liver of DMN and DEN treated female rats is in accord with the report of a decrease of malic dehydrogenase in the rat liver homogenates during administration of the carcinogen acetylaminofluorene (Hou and Rees, 1961). When the authors expressed their results in terms of mitochondrial nitrogen, the difference between treated and control animals disappeared. They concluded that "there is a fall in the mitochondrial population rather than an alteration in the enzymic content of the mitochondria ". A decrease in the number of liver mitochondria was found also in the present experiment and it is likely that the explanation for a decrease in the activity of dehydrogenases in the liver homogenates during nitrosamine or acetylaminofluorene administration is the same.

The concentration of available (free) $\beta$-glucuronidase in liver slices from untreated rats is about one-third of that of total $\beta$-glucuronidase activity as found in homogenates. In order to explain differences found for succinoxidase values in homogenates or in slices of the liver from rats fed *p*-dimethylaminoazobenzene, a different accessibility of parts of the enzymatic system or an intracellular inhibitor was postulated in the intact cells (Hoch-Ligeti, 1947). It is of interest that in liver cells damaged by ischemia, about 30–40 per cent bound enzyme is released from lysosomes (deDuve and Beaufay, 1959). Disorganization of the endoplasmic reticulum, which on homogenization forms the major part of the microsomal fraction (Palade and Siekevitz, 1956), has been observed recently in the liver cells of rats treated with DMN or with 2-fluoreneamine (Gustafsson and Afzelius, 1963). A difference in the intracellular distribution of $\beta$-glucuronidase in normal and malignant cells has been described (Conchie and Levvy, 1959).

Although atypical large cells with bizarre hyperchromic nucleus were already present in the liver at the time of the increase of the $\beta$-glucuronidase concentration their significance with regard to the increase of the enzyme is questionable since another morphological change, namely the formation of groups of large pale cells with vacuolarized cytoplasm occurred at about the same time. These changes might be the expression of reversible cellular damage. It has been suggested (Kerr and Levvy, 1947 ; Levvy, Kerr and Campbell, 1948) that the rise of $\beta$-glucuronidase activity in cells is associated with tissue regeneration following moderate damage rather than with the damage itself. On the other hand, the first frankly neoplastic cells appear in these groups of cells. The $\beta$-glucuronidase activity of the liver increases quite suddenly, weeks before a tumor can be discerned, and it is tempting to assume that at this time the " point of no return " has been reached.

Fishman (1940) showed that the administration of certain toxic substances which are excreted as glucuronides, increase the $\beta$-glucuronidase activity of liver and kidney. The detoxication of substances, which are usually excreted as glucuronides, such as estrogens, might be interfered with in a liver damaged by a carcinogen. The accumulation of these substances could then, in turn, induce an increase in the liver enzyme concentration.

The possibility that the excretion of $\beta$-glucuronidase by the kidney is altered by nitrosamine derivatives should also be considered, particularly since the kidneys were found to be morphologically damaged. Investigations of the excretion of glucuronides in rats fed nitrosamine derivatives are being carried out.

### SUMMARY

The $\beta$-glucuronidase and lactic dehydrogenase (LDH) concentrations of liver, kidney and lung were determined in rats during tumor production by feeding diethylnitrosamine (DEN) or dimethylnitrosamine (DMN). The first tumors occurred on the 113th day in the liver of a DMN treated male and on the 122nd day in the liver of a DEN treated female rat.

In the liver of the treated rats the $\beta$-glucuronidase concentration increased significantly over that of the controls between the 50th and 70th day. In the kidneys the increase was gradual and less pronounced ; in the lung the $\beta$-glucuronidase activity, determined after 130 days, was found to be significantly elevated.

At the time of the increase of $\beta$-glucuronidase activity of the liver, large cells with hyperchromic nuclei and vacuolarized cytoplasm appeared in the central areas of the lobules.

The increase in $\beta$-glucuronidase concentration of the organs was related to the length of treatment with the carcinogen and not to the amount ingested.

The LDH concentration was not increased in the livers of rats during induction of tumors by feeding DEN or DMN. The LDH concentration in the liver tumors was lower than in the surrounding tissue.

This work was aided by Grant P-266-G from the American Cancer Society, Inc.

### REFERENCES

ARGUS, M. F. AND HOCH-LIGETI, C.—(1961) *J. nat. Cancer Inst.*, **27**, 695.
ARGUS, M. F., LEUTZE, C. J. AND KANE, J. F.—(1961) *Experientia*, **17**, 357.

BIERMAN, H. R., HILL, B. R., REINHARDT, L. AND EMORY, E.—(1957) *Cancer Res.*, **17**, 660.
BRUNNGRABER, E. G. AND ABOOD, L. G.—(1960) *J. biol. Chem.*, **235**, 1847.
CONCHIE, J. AND LEVVY, G. A.—(1959) *Nature, Lond.*, **184** (Suppl. 22), 1709.
DEDUVE, C. AND BEAUFAY, H.—(1959) *Biochem. J.*, **73**, 610.
*Idem*, PRESSMAN, B. C., GIANETTO, R., WATTIAUX, R. AND APPELMANS, F.—(1955) *Ibid.*, **60**, 604.
DUBUY, H. G. AND HESSELBACH, M. L.—(1958) *J. nat. Cancer Inst.*, **20**, 403.
FISHMAN, W. H.—(1940) *J. biol. Chem.*, **136**, 229.—(1959) 'Physiopathology of Cancer; A Treatise for Investigators, Physicians and Students'; Homburger, F., ed. New York (Harper), pp. 732–760.
*Idem* AND ANLYAN, A. J.—(1947) *Cancer Res.*, **7**, 808.
GREENSTEIN, J. P.—(1954) 'Biochemistry of Cancer'; 2nd Edition. New York (Academic Press).
GRUNDMANN, E. AND SIEBURG, H.—(1962) *Beitr. path. Anat.*, **126**, 57.
GUSTAFSSON, R. G. AND AFZELIUS, B. A.—(1963) *J. nat. Cancer Inst.*, **30**, 1045.
HESS, R., SCARPELLI, D. G. AND PEARSE, A. G.—(1958) *Nature, Lond.*, **181**, 1531.
HILL, B. R. AND LEVI, C.—(1954) *Cancer Res.*, **14**, 513.
HOCH-LIGETI, C.—(1947) *Ibid.*, **7**, 148.—(1962) *Fed. Proc.*, **21**, 165.
HOLMBERG, B.—(1961) *Cancer Res.*, **21**, 1386.
HOU, C. T. AND REES, K. R.—(1961) *Brit. J. Cancer*, **15**, 624.
HSIEH, K. M., SUNTZEFF, V. AND COWDRY, E. V.—(1956) *Cancer Res.*, **16**, 237.
JACOBSON, K. B. AND NISHIO, K.—(1963) *Ibid.*, **23**, 344.
KERR, L. M. H. AND LEVVY, G. A.—(1947) *Nature, Lond.*, **160**, 463.
LEVVY, G. A., KERR, L. M. H. AND CAMPBELL, J. G.—(1948) *Biochem. J.*, **42**, 462.
MAGEE, P. N. AND HULTIN, T.—(1962) *Ibid.*, **83**, 106.
*Idem* AND FARBER, E.—(1962) *Ibid.*, **83**, 114.
MEISTER, A.—(1950) *J. nat. Cancer Inst.*, **10**, 1263.
NACHLAS, M. M., WALKER, D. G. AND SELIGMAN, A. M.—(1958) *J. biophys. biochem. Cytol.*, **4**, 29.
NOVIKOFF, A. B.—(1961) 'The Cell', Volume 2; Brachet, J. and Mirsky, A. E., ed. New York (Academic Press), pp. 344–345.
PALADE, G. E. AND SIEKEVITZ, P.—(1956) *J. biophys. biochem. Cytol.*, **2**, 171.
SCHOENTAL, R.—(1961) *Nature, Lond.*, **192**, 670.
TALALAY, P., FISHMAN, W. H. AND HUGGINS, C.—(1946) *J. biol. Chem.*, **166**, 757.
VESELL, E. S. AND BEARN, A. G.—(1962) *Proc. Soc. exp. Biol., N.Y.*, **111**, 100.
WARBURG, O., POSENER, K. AND NEGELEIN, E.—(1924) *Biochem. Z.*, **152**, 309.
WEST, M. AND ZIMMERMAN, H. J.—(1958) *Arch. intern. Med.*, **102**, 103.
WROBLEWSKI, F. AND LADUE, J. S.—(1955) *Proc. Soc. exp. Biol., N.Y.*, **90**, 210.